# Synthesis and Antioxidant Effects of Edaravone-Loaded MPEG-2000-DSPE Micelles in Rotenone-Induced PC12 Cell Model of Parkinson’s Disease

**DOI:** 10.3390/nano14231962

**Published:** 2024-12-06

**Authors:** Xin Luo, Linshan Luo, Rong Lai, Yan Li, Hongyan Zhou, Xiting Li

**Affiliations:** 1 Hospital of Stomatology, Sun Yat-sen University, Guangzhou 510055, Chinaluolsh7@mail2.sysu.edu.cn (L.L.); 2 Guangdong Provincial Key Laboratory of Stomatology, Guangzhou 510055, China; 3 Guanghua School of Stomatology, Sun Yat-sen University, Guangzhou 510055, China; 4 Department of Neurology, The First Affiliated Hospital of Sun Yat-sen University, Guangzhou 510080, China

**Keywords:** edaravone, MPEG-2000-DSPE, Parkinson’s disease, bioavailability, antioxidant

## Abstract

Parkinson’s disease (PD) is the second most common neurodegenerative disorder globally that lacks any disease-modifying drug for prevention or treatment. Oxidative stress has been identified as one of the key pathogenic drivers of Parkinson’s disease (PD). Edaravone, an approved free-radical scavenger, has proven to have potential against PD by targeting multiple key pathologies, including oxidative stress, focal mitochondria, and neuroinflammation. However, its bioavailability is potentially restricted due to its poor solubility and short half-life. This study aims to develop a simple and effective drug delivery system for edaravone to enhance its solubility, stability, and bioavailability to improve its neuroprotective efficacy. An MPEG-2000-DSPE-edaravone (MDE) micelle was prepared via solvent evaporation using MPEG-2000-DSPE as a carrier to encapsulate edaravone. The morphology, particle size, zeta potential, chemical structure, and edaravone loading of MDE were evaluated. We then investigated whether such targeted edaravone delivery could provide enhanced neuroprotection. A cell model of PD was established in PC12 cells through exposure to rotenone. The effects of MDE on PC12 cells treated with or without rotenone were evaluated using a cell counting kit-8, calcein acetoxymethyl ester (AM)–propidine iodide (PI) staining, and flow cytometry. Cell migration was evaluated using a wound healing assay. Additionally, the intracellular antioxidant study was performed using an ROS-level-detecting DCFH-DA probe, and the mitochondrial membrane potentials were evaluated using a JC-1 assay. MDE with a drug-loading content of 17.6% and an encapsulation efficiency of 92.8% was successfully prepared. The resultant MDE had a mean particle size of 112.97 ± 5.54 nm with a zeta potential of −42 mV. Cytotoxicity assays confirmed that the MDE (≤200 ug/mL) exhibited promising cytocompatibility with no significant effect on cell viability, cell cycle regulation, or apoptosis levels. Likewise, compared with the free edaravone, no effect on cell migration was noted for MDE. MDE might be able to target edaravone delivery into PC12 cells, increasing the mitochondrial membrane potential and providing a significant local antioxidant effect. The results demonstrated that MPEG-2000-DSPE could be a promising material for enhancing edaravone’s aqueous solubility, stability, and antioxidant effects. MDE could be a potential drug formulation for treating PD and other diseases in which oxidative stress plays a key role in pathogenesis.

## 1. Introduction

Parkinson’s disease (PD) is a neurodegenerative disorder characterized by progressive damage to dopaminergic neurons in the Substantia Nigra. It is the second most common neurodegenerative disease, affecting approximately 1–2% of individuals aged 65 and older [1,2]. Currently, there is no cure for PD, and available treatments primarily aim to alleviate motor symptoms rather than alter disease progression. These treatments include levodopa, dopamine agonists, monoamine oxidase B inhibitors, catechol-O-methyltransferase inhibitors, and deep brain stimulation [3]. However, the efficacy of these therapies tends to decline over time due to the progressive nature of the disease, prompting the search for new therapeutic approaches [4]. Recent studies have increasingly highlighted the role of oxidative stress in PD and other neurodegenerative diseases, such as amyotrophic lateral sclerosis (ALS) and Alzheimer’s disease. A deeper understanding of these mechanisms may aid in the development of novel therapies for neurodegeneration [5].

Edaravone (3-methyl-1-phenyl-2-pyrazolin-5-one, MCI-186, EDR) is a lipophilic compound known for its free-radical scavenging properties, which make it a promising therapeutic agent in neurological disorders. It has demonstrated antioxidant effects by reducing the formation of hydroxyl and superoxide radicals both in vitro and in vivo [6]. Furthermore, edaravone has been shown to inhibit lipid peroxidation, preserve mitochondrial function, and support cellular energy metabolism [7,8,9]. Additionally, edaravone has been approved by the U.S. Food and Drug Administration (FDA) for the treatment of amyotrophic lateral sclerosis (ALS) and is also authorized for the management of ischemic stroke in several Asian countries [10,11]. The current formulation of edaravone is administered intravenously due to its low oral bioavailability [12]. This method requires twice-daily infusions, which can be burdensome for patients [13]. Despite its lipophilic nature, edaravone is a substrate of P-glycoprotein, and its oral absorption is limited by its poor solubility, instability, rapid metabolism, and low intestinal permeability, resulting in an oral bioavailability of only 5.23% [14].

In recent years, nanotechnology has led to significant advancements in drug delivery systems. Nanoparticles offer advantages such as control over the degree of drug loading and release and long-term stability and biocompatibility, providing novel directions for the development of pharmaceutical preparations [15]. Several studies have developed nanoformulations of edaravone, such as ceria [16], solid dispersion [17], lipid-based nanosystems [18], cyclodextrin [14], PLGA-PEG, and boxymethyl cellulose [19] for applications in stroke, inner ear protection, Alzheimer’s disease, and amyotrophic lateral sclerosis. One such nanoparticle carrier is methyl-polyethylene glycol 2000-distearylphosphatidylethanolamine (MPEG-2000-DSPE), a phospholipid–polyethylene glycol conjugate. Known for its excellent biocompatibility and biodegradability, MPEG-2000-DSPE is frequently used to improve drug delivery and enhance release efficiency. Studies have shown that MPEG-2000-DSPE serves as an effective stabilizer for nanocrystals, providing superior colloidal stability and greater surface coverage compared with other stabilizers [20,21,22].

In this study, we investigated the neuroprotective effects of edaravone, focusing on its antioxidant and anti-inflammatory properties. We utilized MPEG-2000-DSPE as a nanocarrier to create a novel formulation, Methyl-PEG2000-DSPE-edaravone (MDE). This new formulation exhibits improved physical properties and demonstrates antioxidant and neuroprotective effects in a rotenone-induced PD cell model. These findings may broaden the potential therapeutic applications of edaravone.

## 2. Materials and Methods

### 2.1. Preparation of MDE

In total, 15 mg of MPEG-2000-DSPE (M3264, TCI, Shanghai, China), 3 mg of edaravone (HY-HY-B0099, MedChemExpress, Shanghai, China), and 1 mL of tetrahydrofuran (THF, 186562, Merck, Taufkirchen, Germany) were added to a 20 mL beaker and stirred at room temperature at 300 r/min. Then, 10 mL of high-purity water was added to the beaker at a rate of 5 s per drop. Stirring continued in the same manner for 24 h after the complete dropwise addition, at which point the THF was volatilized entirely. The resulting liquid was ultra-centrifugated through a 0.22 μm filter, and the resulting aqueous solution was lyophilized at −80 °C to prepare solid powders.

### 2.2. Characterization of MDE

#### 2.2.1. Functional Group and Characteristics of MDE

The functional group characteristics of MDE were evaluated through Fourier transform infrared spectroscopy (INVENIO R, Bruker Optik GMBH, Ettlingen, Germany). After ultrasonic treatment for 10 min, MDE and edaravone solutions at a concentration of 1 mg/mL were dripped on a silicon wafer for natural drying, and then, a gold spraying treatment was carried out. The particle morphology of MDE was detected via scanning electron microscopy (SEM, SEM5000, CIQTEK, Hefei, China). MDE particle sizes in the SEM image were measured by ImageJ software (v2.14.0/1.54f, National Institute of Health, Bethesda, MD, USA).

#### 2.2.2. Zeta Potential

After 1 mg of the MDE and 2 mL of water were added to a 5 mL centrifuge tube, ultrasonic dispersion was performed for 5 min. After that, 1 mL of the supernatant was collected and slowly added to the sample chamber. The zeta potential of the MDE was measured using a Mastersizer ZS90 (Malvern Panalytical, Malvern, UK).

#### 2.2.3. Solubility of MDE

The solubilities of MDE and edaravone were determined via UV–visible spectrophotometry. Excessive MDE and edaravone were added to deionized water. The mixture was stirred at room temperature for 24 h and centrifuged at 12,000 rpm for 5 min. The supernatants were collected, filtered through 0.22 μm filters, and diluted 10 times to measure their absorbance at a wavelength of 240 nanometers using a UV–visible spectrophotometer (Nanodrop, Thermo Scientific, Waltham, MA, USA). The concentrations of edaravone and MDE were determined based on the absorbance curve of their gradient concentrations.

#### 2.2.4. Drug-Loading Content and Encapsulation Efficiency of MDE

To measure drug loading and drug-loading efficiency, 1 mg of MDE was accurately weighed and dissolved in 1 mL of deionized water. The edaravone mass was evaluated by detecting the OD value at 240 nm and calculated according to the standard curve of edaravone mentioned above. Drug loading was calculated according to the following equation:

$$\mathrm{drug}\;\mathrm{loading}\;\left(\%\right)=\frac{{\mathrm{mass}}_{\mathrm{edaravone}\;\mathrm{in}\;\mathrm{MDE}}}{{\mathrm{mass}}_{\mathrm{MDE}}}\times 100\%$$

Drug loading efficiency was calculated according to the following equation:

$$\mathrm{drug}\;\mathrm{loading}\;\mathrm{efficiency}\;\left(\%\right)=\frac{{\mathrm{mass}}_{\mathrm{edaravone}\;\mathrm{in}\;\mathrm{MDE}}}{{\mathrm{mass}}_{\mathrm{edaravone}\;\mathrm{added}}}\;\times 100\%$$

### 2.3. Antioxidant Activity of MDE

The DPPH method was used to measure the antioxidant activity of MDE. MDE was added to a 4 μg/mL DPPH/ethanol solution at concentrations of 3.125, 12.5, 50, 200, and 500 μg/mL. Then, a 4 μg/mL DPPH/ethanol solution and ethanol were used as controls. The solutions were set at 37 °C in the dark for 30 min, and the OD values at 517 nm were detected through a microplate reader (Epoch 2 BioTek, Agilent, Winooski, VT, USA). The antioxidative rates were calculated according to the following equation:

$$Radical\;scavenging\;ratio\;(\%)=\frac{{OD}_{DPPH}-{OD}_{sample}}{{OD}_{DPPH}-{OD}_{blank}}\times 100\%$$

*OD_DPPH_*, *OD_sample_*, and *OD_blank_* were identified as the OD value of the 4 μg/mL DPPH/ethanol solution, the MDE solutions, and the ethanol solutions at 517 nm, respectively.

### 2.4. Cell Culture and Grouping

Differentiated PC12 cells—a rat neuronal cell line derived from pheochromocytoma cells—were purchased from the Cell Library of the Chinese Academy of Science (Shanghai, China). PC12 cells were cultured in MEM medium with 10% horse serum, 5% fetal bovine serum, and 1% Penicillin–Streptomycin at 37 °C, 95% relative humidity.

The cells were treated differently in different experiments. For the cytotoxicity test, PC12 cells were treated with various MDE gradient concentrations (0, 3.125, 12.5, 50, and 200 μg/mL) for 24 or 72 h. To evaluate the protective effects of MDE on cell injury, PC12 cells were treated with 1 μM of rotenone (HY-B1756, MedChemExpress, China) in the presence or absence of MDE (12.5 and 50 μg/mL with available edaravone concentrations of 2.1 and 8.4 μg/mL, respectively) or edaravone (2.1 and 8.4 μg/mL). To determine cell biological activity and cellular antioxidant activity, PC12 cells were treated with 1 μM of rotenone for 24 h and subsequently co-cultured with different concentrations of MDE or edaravone for another 48 h. Six groups were set up, including a blank group (no treatment), a rotenone group (1 μM rotenone), a rotenone + MDE12.5 group (1 μM rotenone and 12.5 μg/mL MDE), a rotenone + MDE12.5 group (1 μM rotenone and 12.5 μg/mL MDE), a rotenone + EDR2.1 group (1 μM rotenone and 2.1 μg/mL edaravone), and a rotenone + EDR8.4 group (1 μM rotenone and 8.4 μg/mL edaravone).

### 2.5. In Vitro Cytotoxicity of MDE

#### 2.5.1. Cell Counting Kit-8 (CCK-8)

Cell viability was determined using a CCK-8 kit (ST1007, Saint-Bio, Shanghai, China). A 100 μL cell suspension (1 × 10^5^ cells/mL) was inoculated into 96-well plates and incubated with rotenone or MDE at 37 °C for 24 or 72 h, respectively. After 1 h of incubation with the CCK-8 solution, the absorbance at 450 nm was recorded with a microplate reader (Epoch 2, BioTek, USA), and cell viability was calculated according to the following equation:

$$\mathrm{Relative}\;\mathrm{cellular}\;\mathrm{viability}\;\left(\%\right)=\frac{{\mathrm{OD}}_{\mathrm{sample}}-\;{\mathrm{OD}}_{\mathrm{blank}}}{{\mathrm{OD}}_{\mathrm{ctrl}}-\;{\mathrm{OD}}_{\mathrm{blank}}}\times \;100\%$$

#### 2.5.2. Living Cell Detection

A calcein acetoxymethyl ester (AM)–propidine iodide (PI) working solution was obtained by mixing calcein AM and PI solutions included in the calcein PI kit (C2015, Beyotime, Shanghai, China). The cells were inoculated with the calcein AM/PI working solution at 37 °C in the dark for 30 min. At the end of the incubation period, the staining was observed under a confocal laser scanning microscope (LSM980, Zeiss, Oberkochen, Germany).

#### 2.5.3. Cell Cycle

The cell cycle was determined using a cell cycle kit (R21806, Saint-Bio, Shanghai, China). Briefly, cells were digested with trypsin, collected in centrifuge tubes, and centrifuged at 1000 g for 5 min. 1 mL cold phosphate buffer saline (PBS, AC13317, Acmec, Shanghai, China) was used to resuspend cells, and cold 70% ethanol (1 mL) was used to fix cells. After being fixed at 4 °C for 2 h, the cells were washed with PBS and then stained with DAPI in the dark at 4 °C for 30 min. Flow cytometry (Beckman CytoFLEX, Beckman Coulter, Brea, CA, USA) was used to detect red fluorescence at an excitation wavelength of 488 nm.

#### 2.5.4. Cell Apoptosis

Cell apoptosis was assessed using a commercial apoptosis detection kit (RK05875, ABclonal, Wuhan, China). Initially, the cells were washed with ice-cold PBS and resuspended in binding buffer at a concentration of 1 × 10^6^ cells/mL. A 100 μL aliquot of the cell suspension was then stained with APC and DAPI, followed by incubation in the dark for 15 min. Apoptotic cells were quantified using flow cytometry (Beckman CytoFLEX, Beckman Coulter, Brea, CA, USA).

#### 2.5.5. Cell Migration

Cell migration was evaluated using a wound healing assay. Cells were grown to confluence in a 6-well plate, and a scratch was made in the monolayer using a sterile pipette tip. The medium was replaced with fresh culture medium containing 1 μM of rotenone and MDE at various concentrations. The migration of cells into the wounded area was observed over time.

### 2.6. Cellular Antioxidant Activity

Cellular antioxidant activity was determined by measuring reactive oxygen species (ROS) levels in PC12 cells using ROS-sensitive probes. PC12 cells were seeded in a 24-well plate and exposed to 1 μM rotenone for 24 h. Then, the medium was replaced with fresh medium containing 1 μM of rotenone and various concentrations of MDE, and the cells were incubated for another 48 h. The cells were subsequently stained with dichlorodihydrofluorescein diacetate (DCFH-DA) and analyzed using a confocal laser scanning microscope (LSM980, Zeiss, Oberkochen, Germany). The average fluorescence intensities were quantified using ImageJ software.

### 2.7. Measurement of Mitochondrial Membrane Potential

Mitochondrial membrane potential was assessed using the JC-1 assay kit. PC12 cells were seeded in a 96-well plate at a density of 1 × 10^4^ cells per well and treated with 1 μM rotenone for 24 h. Afterward, the medium was replaced with fresh culture medium containing 1 μM rotenone and MDE at various concentrations. The cells were incubated for an additional 48 h and then stained with JC-1 dye according to the manufacturer’s protocol. Mitochondrial membrane potential was observed using a confocal laser scanning microscope (LSM980, Zeiss, Oberkochen, Germany).

### 2.8. Statistical Analyses

All data are expressed as mean ± standard deviation (SD). One-way analysis of variance (ANOVA) was performed to compare differences between multiple groups, followed by a Bonferroni post hoc test. Statistical analyses were conducted using SPSS 16.0 (IBM Corp., Poughkeepsie, NY, USA), with a p-value of less than 0.05 considered statistically significant.

## 3. Results

### 3.1. Preparation of MDE

**Figure 1:**
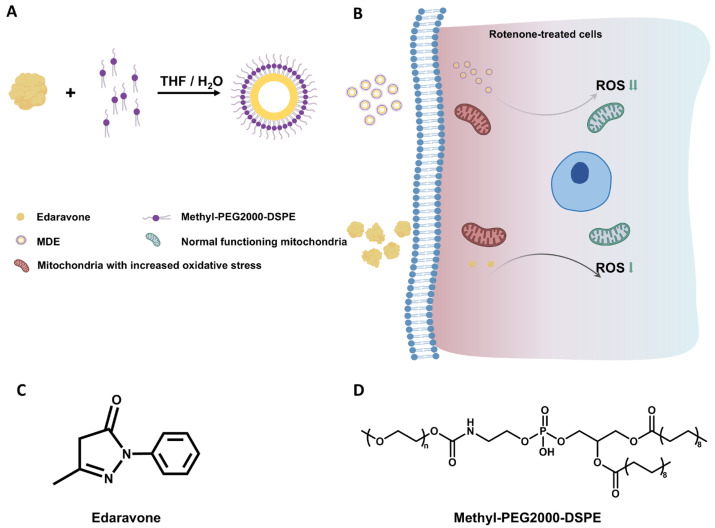
Schematic diagram of formation and intracellular antioxidation of MPEG-2000-DSPE-edaravone (MDE). (**A**) Schematic structure of edaravone and methyl-PEG2000-DSPE self-assembly into micelles. (**B**) Edaravone and MDE targeting the focal-cell-attenuated over-generation of intracellular ROS. (**C**) Chemical structure of edaravone. (**D**) Chemical structure of methyl-PEG2000-DSPE.

MDE was prepared by encapsulating edaravone with MPEG-2000-DSPE using a solvent evaporation method. A schematic diagram of the formation of MDE is illustrated in Figure 1A. Figure 1B shows that edaravone and MDE targeted the focal cells with increased oxidative stress and effectively alleviated the over-generation of intracellular ROS.

The chemical structures of edaravone and MPEG-2000-DSPE are illustrated in Figure 1C,D.

### 3.2. Characterization of MDE

**Figure 2:**
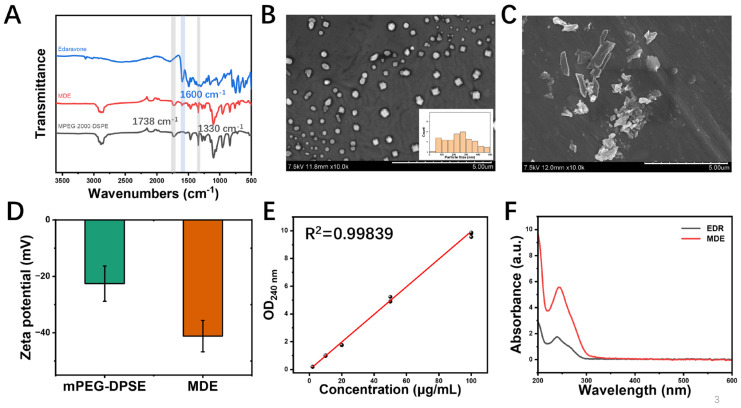
Physical properties of MPEG-2000-DSPE-edaravone (MDE) micelles. (**A**) Fourier transform infrared spectroscopy of MDE, edaravone, and MPEG-2000-DSPE. (**B**) Scanning electron microscope image and the distribution of particle sizes of MDE; scale bar is 5 μm. (**C**) Scanning electron microscope image of edaravone (EDR); scale bar is 5 μm. (**D**) Zeta potential map of MDE and MPEG-2000-DSPE. (**E**) Standard curve of MDE. (**F**) Aqueous solubility curve of MDE (red) and edaravone (black).

Functional groups, particle size, zeta potential, morphology, and solubility are illustrated in Figure 2. The functional groups of MDE were evaluated using Fourier transform infrared spectroscopy. Figure 2A shows that the FTIR spectra of MDE, edaravone, and MPEG-2000-DSPE were loaded individually. MDE could form mixed micelles with uniform particle size distribution. The morphology of MDE and edaravone was observed via SEM. The MDE micelles prepared had a particle size of 112.97 ± 5.54 nm with a spherical shape and formed mixed micelles with uniform distribution in an aqueous solution (Figure 2B, Table S1). The SEM images show that the edaravone aggregates had a conventionally large size and different shapes (Figure 2C). Figure 2D shows that the zeta potential value of MDE was −41.33 ± 0.611 mV, higher than methyl-PEG2000-DSPE (−21.40 ± 2.955 mV). In addition, the linear standard curve (Figure 2E) and alterative dissolution curve (Figure 2F) indicate the enhanced stability and biocompatibility of the MDE micelles compared with edaravone. The drug-loading content was up to 17.6%, and the encapsulation efficiency was up to 92.8%. The small particle size and high drug loading of MDE could be advantageous for drug delivery and penetration, especially for crossing the blood–brain barrier.

### 3.3. In Vitro Cytotoxicity of MDE

**Figure 3:**
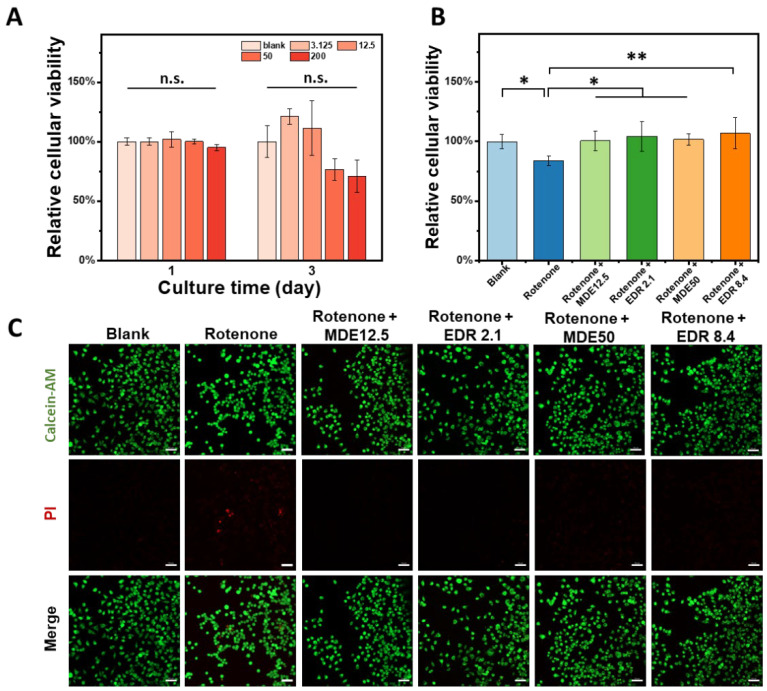
Cytotoxicity of MPEG-2000-DSPE-edaravone (MDE). (**A**) PC12 cells were treated with gradient concentrations of MDE (0, 3.125, 12.5, 50, and 200 μg/mL) for 72 h, and then cell viability was determined using CCK-8. For (**B**,**C**), a cell model of Parkinson’s disease was established in PC12 cells through exposure to rotenone (1 μM) for 24 h, which were then treated with MDE (12.5 and 50 μg/mL) or edaravone (2.1 and 8.4 μg/mL) for another 48 h. (**B**) Cell viability was determined using CCK-8. (**C**) Cells were stained with calcein acetoxymethyl ester (AM) and propidine iodide (PI) to evaluate cell living. Each experiment was repeated at least six times. Scale bars are 50 μm. * *p* < 0.05 versus blank group. ** *p* < 0.01 versus blank group.

Firstly, we evaluated the cytotoxicity of MDE in rat pheochromocytoma cell line PC12. Figure 3A shows that the gradient concentrations of MDE (0, 3.125, 12.5, 50, and 200 μg/mL) have no significant effect on PC12 cell viability. Next, we treated the PC12 cells with rotenone to mimic Parkinson’s injury, observing a significant decrease in cell viability that was effectively reversed after the continued administration of MDE or edaravone (Figure 3B). Subsequently, we determined the number of living and dead cells by means of double staining with calcein AM (green) and PI (red). This revealed that rotenone increased the number of dead PC12 cells; this effect was partially reversed by MDE and edaravone (Figure 3C).

### 3.4. Neuroprotective Effects of MDE in Parkinson’s Disease Cell Model

**Figure 4:**
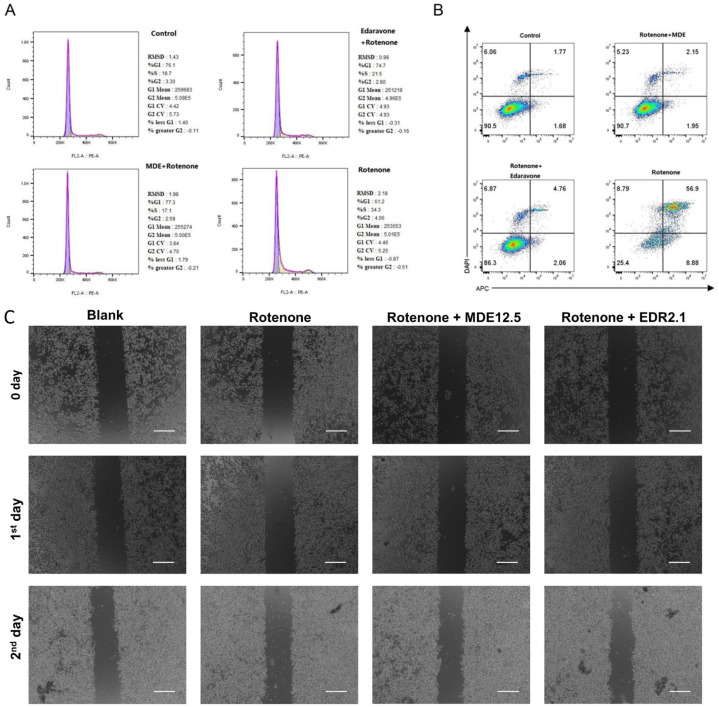
Neuroprotective effects of MPEG-2000-DSPE-edaravone (MDE) on Parkinson’s disease cell model. A cell model of Parkinson’s disease was established in PC12 cells through exposure to rotenone (1 μM), and the reversible effects of MDE and edaravone were investigated. PC12 cells were divided into four groups: blank group (no treatment), rotenone group (1 μM rotenone), rotenone + MDE group (1 μM rotenone and 12.5 μg/mL MDE), and rotenone + EDR group (1 μM rotenone and edaravone solution). (**A**) Cell cycle determined via flow cytometry. (**B**) Cell apoptosis determined via flow cytometry. (**C**) Cell migration determined via wound healing assay. Each experiment was repeated at least six times. Scale bars are 200 μm.

A Parkinson’s disease cell model was induced in PC12 cells by treating them with 1 μM of rotenone, which is a commonly used method for evaluating neuroprotection in vitro [19]. The potential neuroprotective effects of MDE and edaravone were assessed through various assays, including cell cycle analysis, apoptosis detection, and migration studies. Flow cytometry was utilized to evaluate the impact of MDE micelles on PC12 cell apoptosis. Treatment with rotenone reduced the proportion of cells in the S-phase while increasing the percentage of apoptotic cells, compared with the untreated controls (Figure 4A,B). Notably, both edaravone and MDE were able to reverse the effects of rotenone, restoring the cell cycle distribution and reducing apoptosis, with MDE showing a stronger protective effect than edaravone (Figure 4A,B). Furthermore, the wound healing assay revealed that, similar to edaravone, MDE had no significant impact on the migration of PC12 cells exposed to rotenone (Figure 4C).

### 3.5. Antioxidant Effects of MDE in Parkinson’s Disease Cell Model

**Figure 5:**
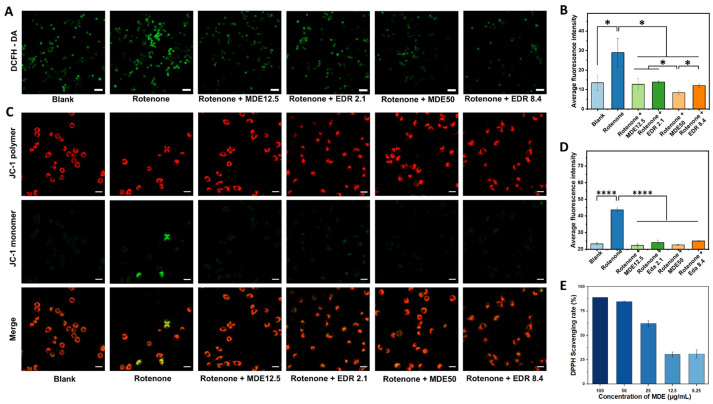
Antioxidant effects of MPEG-2000-DSPE-edaravone (MDE) in Parkinson’s disease cell model. In the PD cell model, the logarithmic-growth-stage PC12 cells were treated with 1 μM rotenone for 24 h and further co-cultured with different concentrations of MDE or edaravone for another 48 h. Representative images were taken after incubating the vehicle medium, free edaravone, or MDE for 48 h. The intracellular reactive oxygen species (ROS) level was detected using a DCFH-DA fluorescent probe. (**A**) DCFH-DA staining. (**B**) Average fluorescence intensity of DCFH-DA. The mitochondrial membrane potentials were measured using the JC-1 assay kit. (**C**) JC-1 staining (mitochondrial membrane potentials). (**D**) Average fluorescence intensity of JC-1 monomer. (**E**) The antioxidant activity of MDE was evaluated using a DPPH radical scavenging assay. Data are presented as the mean ± SD of six independent experiments. * *p* < 0.05, **** *p* < 0.0001 versus control group. Scale bars in A are 50 μm. Scale bars in C are 20 μm.

Oxidative stress is a key factor contributing to rotenone-induced toxicity in PC12 cells. Rotenone inhibits mitochondrial complex I, leading to the generation of reactive oxygen species (ROS). As shown in Figure 5A,B, rotenone treatment resulted in significant ROS overproduction in PC12 cells. However, both edaravone and MDE notably reduced ROS levels in these cells. This reduction in ROS production could be an important mechanism underlying the neuroprotective effects of MDE and edaravone in rotenone-treated PC12 cells.

The mitochondrial membrane potential was measured using the JC-1 assay kit, which could detect changes in mitochondrial health. Red fluorescence from JC-1 aggregates indicates a high mitochondrial membrane potential, while green fluorescence from JC-1 monomers suggests a reduced potential, indicative of early apoptosis. Figure 5C,D demonstrate that after rotenone treatment, PC12 cells exhibited strong green fluorescence in the cytoplasm, signaling that the cells were in the early stages of apoptosis. However, when these cells were treated with various concentrations of MDE and edaravone, only faint green fluorescence was observed, suggesting that both compounds were able to partially restore mitochondrial function, even at low MDE concentrations (12.5 μg/mL).

In Figure 5E, the antioxidant and radical scavenging activities of MDE micelles were shown to be comparable to free edaravone. Rotenone exposure significantly reduced cell survival, induced apoptosis, decreased mitochondrial membrane potential, and elevated ROS levels compared with the control group. These detrimental effects were substantially mitigated by MDE and edaravone treatments, highlighting their potential to prevent oxidative damage in the mitochondria of PC12 cells. These findings support the possibility of MDE as a promising therapeutic option for Parkinson’s disease.

## 4. Discussion

Edaravone is widely recognized for its properties as a free-radical scavenger, anti-apoptotic agent, and anti-inflammatory and anti-cytokine mediator, showing therapeutic benefits in several diseases. Developed by Mitsubishi Tanabe Pharma Corporation (Osaka, Japan) in 2001, edaravone is commercially available as Radicava^®^ and Radicut^®^, both intravenous formulations approved for treating amyotrophic lateral sclerosis and cerebral infarction [23]. Moreover, edaravone has demonstrated neuroprotective effects in other neurodegenerative conditions, including Parkinson’s disease, Alzheimer’s disease, and meningitis [7,24,25]. However, its clinical use is limited by its poor oral bioavailability, which results from its low aqueous solubility, instability, rapid metabolism, and limited intestinal permeability [14]. Additionally, edaravone has a short half-life and faces challenges in crossing the blood–brain barrier (BBB), further restricting its therapeutic efficacy and stability [26]. To overcome these limitations, efficient drug delivery systems are urged to be developed for enhancing the bioavailability and cerebral circulation time of edaravone. In this study, we present a novel formulation of edaravone, MDE, designed to improve its pharmacokinetic properties and extend its therapeutic effects.

The physical properties of MDE, compared to free edaravone, show remarkable improvements in neuroprotection, including enhanced aqueous solubility and stability.

It was quite clear that MDE’s physical properties for neuroprotection were improved compared with free edaravone; its aqueous solubility and stability were enhanced by employing MPEG-2000-DSPE as a nanocarrier. The reasons are as follows. MPEG-2000-DSPE offers several advantages for oral drug delivery due to its unique characteristics, including a tunable pore size, high surface area, adjustable surface chemistry, and a favorable morphological structure. Incorporating drugs into MPEG-2000-DSPE allows for the modification of drug pharmacokinetics, protection from adverse environmental factors, and prevention of drug crystallization during storage. The repeat ethylene glycol units in PEG confer both hydrophilicity and flexibility, forming a stable hydration layer through hydrogen bonding with surrounding water molecules [27,28]. This structure helps protect encapsulated edaravone from rapid metabolism by plasma enzymes and other substances. Furthermore, the use of PEG–lipid conjugates in nanocarriers reduces the need for small liposomes to achieve prolonged circulation times [29]. These physicochemical properties enhance drug solubility and stability, while the PEGylated surface also minimizes non-specific protein adsorption, including opsonins, which could lead to faster clearance from the bloodstream [30,31].

MDE nanoparticles exhibited a mean size of 112.97 ± 5.54 nm and a spherical morphology, in contrast to traditional edaravone formulations, which tend to have larger sizes and more variable shapes. This size range aligns with other reported edaravone nanocarriers. For instance, Lu et al. developed PLGA-PEG-based nanoparticles for intranasal delivery of edaravone, achieving a size of less than 100 nm, which resulted in high drug loading, improved solubility, and better shelf-life stability [19]. Similarly, Parikh et al. fabricated lipid–polymer hybrid nanoparticles for plasmid DNA delivery using PLGA, DC-cholesterol, and mPEG2000-DSPE, resulting in particles approximately 150 nm in size with a spherical shape [21,22]. These studies suggest that the size and morphology of edaravone nanocarriers are influenced by the materials used and their ratios in the formulation. Moreover, it has been noted that smaller nanoparticles are more effective at utilizing the enhanced permeability and retention (EPR) effect, which helps the drug passively target brain lesions and improve neuroprotective outcomes [32]. Smaller particles also facilitate BBB penetration [33], further enhancing the potential therapeutic efficacy of MDE. Notably, MDE demonstrated uniform distribution and efficient penetration into the brain, specifically preventing the degeneration of dopamine neurons, thereby improving the therapeutic efficacy of edaravone. Given its smaller size and rod-like morphology, we hypothesize that MDE may outperform spherical nanoparticles in terms of membrane penetration and BBB crossing.

We synthesized MDE with a zeta potential of −42 mV. Zeta potential is an important indicator for assessing the stability of particle dispersion. A higher zeta potential signifies stronger electrostatic repulsion between particles, which reduces their tendency to aggregate [34]. For example, Mozafari et al. developed glutathione-conjugated poly (methacrylic acid) nanogels for edaravone delivery, resulting in nanoparticles with a zeta potential of −25 mV [35]. The lower zeta potential observed in our MDE formulation suggests that it exhibits greater stability and a reduced propensity for aggregation. Moreover, because most blood cells and other biological components are negatively charged, negatively charged nanoparticles like MDE are less likely to interact non-specifically with these components [36], potentially enhancing the circulation time and effectiveness of the drug. The MDE formulation demonstrated a drug loading of 17.6% and an encapsulation efficiency of 92.8%, comparable to other nanoparticle-based delivery systems [19,31].

PD is a progressive neurodegenerative disorder, the exact mechanisms of which remain unclear. It is thought to be driven by a combination of genetic predisposition, environmental factors, mitochondrial dysfunction, and oxidative and nitrosative stress [37,38]. Reactive oxygen species (ROS), including superoxide anions, can interact with nitric oxide to form peroxynitrite, a potent oxidant involved in the pathogenesis of neurodegenerative diseases [39]. In PD, oxidative stress and excessive ROS production contribute to the death of dopaminergic neurons [40]. Therefore, targeting ROS production may halt the progression of PD and protect neurons from degeneration [41]. Although there is no disease-modifying treatment for PD, available pharmacotherapies can help alleviate symptoms. Edaravone, a well-known free-radical scavenger and antioxidant, has shown promise as a therapeutic agent in diseases driven by oxidative stress. The mechanisms through which edaravone exerts its effects in PD have been well documented. These include direct scavenging of harmful ROS, such as hydroxyl, peroxyl, and superoxide radicals; enhancing antioxidant enzyme activity; donating electrons to free radicals and receptors; and modulating intracellular ion concentrations [42]. However, edaravone’s clinical application is limited by its poor bioavailability and other challenges, underscoring the need for biocompatible carriers to optimize its delivery and targeting.

In this study, we developed an in vitro PD model using PC12 cells treated with rotenone, which inhibits mitochondrial complex I and induces ROS production. The primary neuroprotective effects of MDE in this model were as follows: (1) reduction in rotenone-induced cell death and apoptosis; (2) prevention of cell cycle arrest caused by rotenone exposure; (3) inhibition of ROS overproduction; and (4) protection of mitochondria from rotenone-induced damage in PC12 cells. Notably, MDE was effective at a low concentration (12.5 μg/mL), equivalent to 2.1 μg/mL of free edaravone. These results suggest that encapsulating edaravone within MPEG-2000-DSPE nanoparticles enhances its neuroprotective properties, potentially offering a more effective approach for PD treatment.

Rotenone is known to induce neurotoxicity by disrupting cellular energy production and increasing the generation of reactive oxygen species (ROS), which subsequently hampers cell replication and promotes cell death in PC12 cells [43]. In contrast, MDE has been shown to stimulate cell replication and reduce apoptosis in rotenone-treated PC12 cells, which aligns with previous findings that edaravone can protect neurons in a rotenone-induced Parkinson’s disease (PD) model [44,45]. Moreover, it has been reported that MPEG-2000-DSPE enhances the systemic circulation time and improves drug delivery to targeted cells [46,47]. In our study, MDE demonstrated a more pronounced effect than edaravone in modulating the cell cycle and preventing apoptosis in rotenone-treated PC12 cells, which is likely due to the encapsulation of edaravone within the nanocarrier.

Mitochondria are well known for generating ROS under high membrane potential conditions [48]. In PD models, edaravone has been shown to mitigate oxidative damage in PC12 cells exposed to Aβ25-35, as well as in dopaminergic neurons treated with rotenone and 6-hydroxydopamine (6-OHDA) [8,49]. Edaravone also protects PC12 cells from apoptosis and ROS overproduction induced by MPP (+) [50]. Similarly, edaravone has been reported to shield neurons from glutamate toxicity, reduce ROS generation, and alleviate neurotoxicity resulting from hypoxia–acidosis/reoxygenation [51,52]. For example, Qunqun Bao and colleagues demonstrated that loading edaravone into ceria nanoparticles significantly enhanced its antioxidant properties, providing effective protection against stroke [21]. In our study, rotenone-treated PC12 cells exhibited elevated levels of ROS and mitochondrial membrane potential. However, treatment with both edaravone and MDE alleviated mitochondrial dysfunction, restoring mitochondrial membrane potential. These results suggest that MDE offers robust protection against rotenone-induced toxicity in an in vitro PD model, thus providing deeper insight into edaravone’s neuroprotective mechanisms. Additionally, future studies should explore the development of smart drug delivery systems, capable of responsive release triggered by ROS, to further assess the feasibility of MDE for biomedical applications [53,54].

## 5. Conclusions

This study indicated that MPEG-2000-DSPE is a promising material for enhancing aqueous solubility, stability, and antioxidant properties of edaravone. MDE demonstrated significantly stronger neuroprotective and antioxidant effects compared with free edaravone. However, further optimization of the MDE formulation process is needed to improve drug stability, and additional in vivo studies are required to validate the therapeutic efficacy of MDE. MDE shows promising potential as a drug candidate for the treatment of PD and other diseases characterized by oxidative stress.

## Data Availability

The data are contained within the article.

## Supplementary Materials

The following supporting information can be downloaded at: https://www.mdpi.com/article/10.3390/nano14231962/s1, Table S1: The particle size, zeta potential, and polydispersity index of MDE with different ratios of edaravone and MPEG-2000-DSPE; Figure S1: Critical micelle concentration of MPEG-2000-DSPE measured via dynamic light scattering method. Reference [55] is cited in the supplementary materials.
