# Supplementary material for: Synthesis and Antioxidant Effects of Edaravone-Loaded MPEG-2000-DSPE Micelles in Rotenone-Induced PC12 Cell Model of Parkinson’s Disease
Source: Nanomaterials (Basel). 2024 Dec 6;14(23):1962. doi: 10.3390/nano14231962 (PMC11643961; doi:10.3390/nano14231962)
Supplement: Supplementary file 1 [file nanomaterials-14-01962-s001.zip › nanomaterials-3329558-supplementary.pdf]

Supplementary material

S.1.Characterization of MDE

Particle size, zeta potential, and polydispersity index of the MDE micelles with different edaravone/MPEG-2000-DSPE ratios are illustrated in Table S1 and Figure S1.

In this study, to increase the drug payload while maintaining the small size and stability, different edaravone /MPEG-2000-DSPE ratios (1:10, 1:5, 2:5) were investigated for the preparation of MDE. The particle size, zeta potential, and polydispersity index (PDI) of the MDE micelles were measured using Elitesizer (Brookhaven, USA) with dynamic light scattering (DLS). As Table S1 shows, when the drug/stabilizer ratio was 1:5, the MDE micelle had the smallest particle size and the lowest zeta potential. Therefore, the edaravone /MPEG-2000-DSPE ratio of 1:5 was chosen for the subsequent preparation of MDE.

Table S1. The particle size, zeta potential, and polydispersity index of MDE with different ratios of edaravone and MPEG-2000-DSPE.

Edaravone: MPEG-2000-DSPE (m: m)	Particle size (nm)	Zeta potential (mV)	PDI
1:10	140.95±4.39	-37.17±0.71	0.228±0.026
1:5	112.97±5.54	-41.60±5.00	0.275±0.021
2:5	151.40±14.76	+0.15±5.11	0.303±0.024

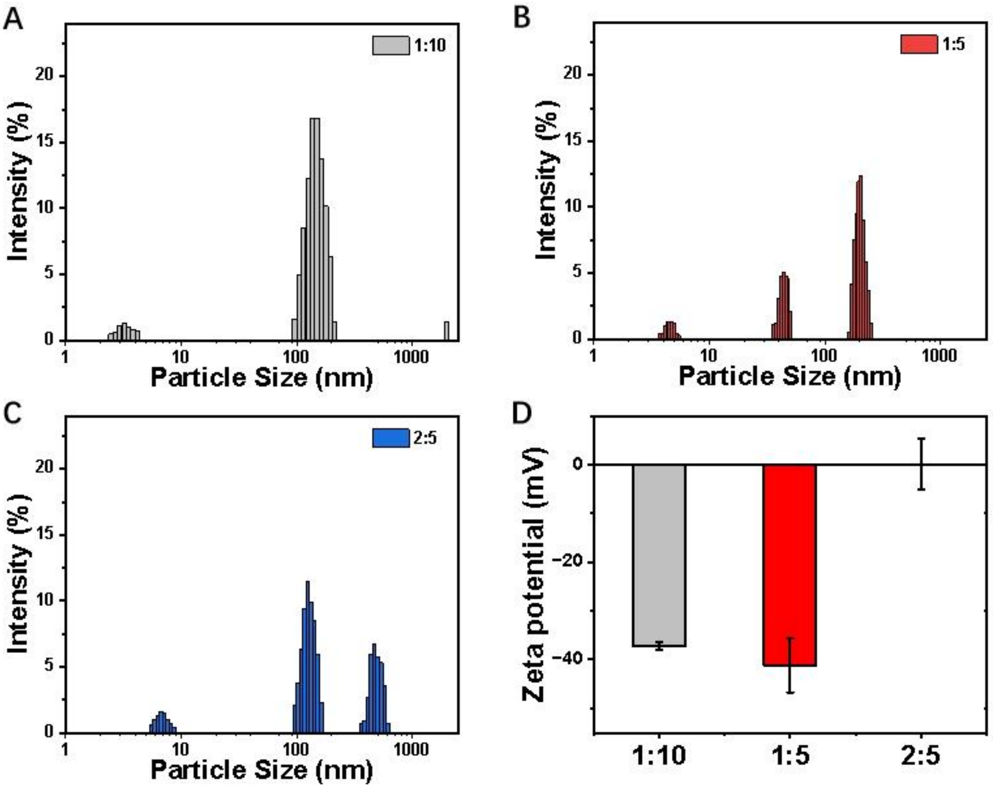

Figure S1. The particle sizes and zeta potentials of MDE with varied ratios of edaravone and MPEG-2000-DSPE.

For (A-C): The distributions of the MDE particle sizes with varied ratios of edaravone and MPEG-2000-DSPE. (A) The edaravone /MPEG-2000-DSPE ratio is 1:10. (B) The edaravone /MPEG-2000-DSPE ratio is 1:5. (C) The edaravone /MPEG-2000-DSPE ratio is 2:5. (D) The zeta potentials of MDE with varied ratios of edaravone and MPEG-2000-DSPE.

S.2. CMC of the MPEG-2000-DSPE

The critical micelle concentration of MPEG-2000-DSPE was determined via the dynamic light scattering method, as previously reported in the literature [1]. A MPEG-2 000-DSPE aqueous solution at different gradient concentrations was prepared, and the number of particles in the solution was measured using a Zetasizer (Brookhaven, USA) with a dynamic light scattering method. A linear fitting was conducted between derived count rates and the concentrations of MPEG-2000-DSPE aqueous solutions. The CMC of the MPEG-2000-DSPE was quantified as the concentration of intersection of the fitting curve and the baseline. As Figure S2 shows, the critical micelle concentration (CMC) of MPEG-2000-DSPE were measured using Elitesizer (Brookhaven, USA) with dynamic light scattering (DLS). In this study, the CMC of MPEG-2000-DSPE was 0.032 mg/mL.

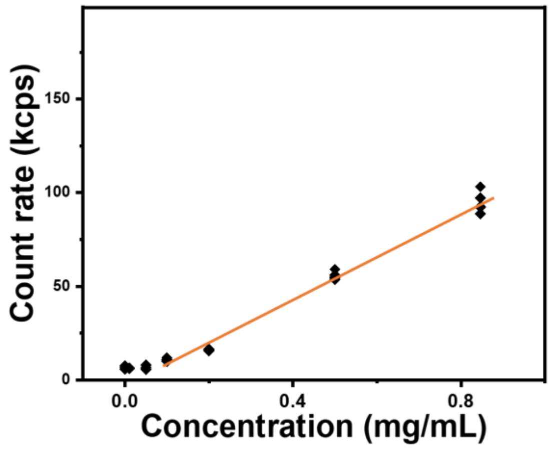

Figure S2. Critical micelle concentration of MPEG-2000-DSPE measured via dynamic light scattering method (n=5).

Reference

Degrand, L.; Garcia, R.; Urion, K.C.; Guiga, W. Dynamic light scattering for the determination of linoleic acid critical micelle concentration. Effect of pH, ionic strength, and ethanol. *J. Mol. Liq.* **2023**, 388, 122670.
